# Estimates of Outbreak Risk from New Introductions of Ebola with Immediate and Delayed Transmission Control 

**DOI:** 10.3201/eid2108.150170

**Published:** 2015-08

**Authors:** Damon J.A. Toth, Adi V. Gundlapalli, Karim Khader, Warren B.P. Pettey, Michael A. Rubin, Frederick R. Adler, Matthew H. Samore

**Affiliations:** University of Utah, Salt Lake City, Utah, USA (D.J.A. Toth, A.V. Gundlapalli, K. Khader, W.B.P. Pettey, M.A. Rubin, F.R. Adler, M.H. Samore);; US Department of Veterans Affairs Salt Lake City Health Care System, Salt Lake City (D.J.A. Toth, A.V. Gundlapalli, K. Khader, W.B.P. Pettey, M.A. Rubin, M.H. Samore).

**Keywords:** Ebola, Ebola virus, viruses, transmission, outbreaks, mathematical model, risk assessment, public health, surveillance, Africa, Guinea, Sierra Leone, Liberia

## Abstract

While the ongoing Ebola outbreak continues in the West Africa countries of Guinea, Sierra Leone, and Liberia, health officials elsewhere prepare for new introductions of Ebola from infected evacuees or travelers. We analyzed transmission data from patients (i.e., evacuees, international travelers, and those with locally acquired illness) in countries other than the 3 with continuing Ebola epidemics and quantitatively assessed the outbreak risk from new introductions by using different assumptions for transmission control (i.e., immediate and delayed). Results showed that, even in countries that can quickly limit expected number of transmissions per case to <1, the probability that a single introduction will lead to a substantial number of transmissions is not negligible, particularly if transmission variability is high. Identifying incoming infected travelers before symptom onset can decrease worst-case outbreak sizes more than reducing transmissions from patients with locally acquired cases, but performing both actions can have a synergistic effect.

## Abstract 2

Identifying incoming patients can have a larger risk-reduction effect than efforts to reduce transmissions from identified patients.

The ongoing Ebola outbreak in West Africa, thought to have begun from a single index case in Guinea in December 2013 (*1*), has produced thousands of cases in Guinea, Sierra Leone, and Liberia (*2*). This Ebola outbreak is the largest and most widespread since the Ebola virus was discovered in 1976 (*3*), and the probability of international spread outside of West Africa is not negligible (*4*). By late April 2015, the virus had been introduced by 7 infected people traveling during their incubation or symptomatic periods to a country other than Guinea, Sierra Leone, or Liberia. Of these 7 cases, 1 led to an outbreak with 19 transmissions in Nigeria (5*,*^6^); 1 led to 2 transmissions in the United States (*7*,*8*); 1 led to 7 transmissions in Mali (*9*,*10*); and 4 led to no transmissions in Mali (*11*), Senegal (*12*), the United States (*13*), and the United Kingdom (*14*). Additionally, 20 persons who acquired infection in Africa were transferred to the United States and several European countries for treatment (*15*), leading to 1 transmission in Spain (*16*).

Although none of these introductions led to a long chain of transmissions, even a small outbreak in a new country can cause societal disruption and disproportionate costs (*17*). Furthermore, how likely it is that an introduced case will lead to a substantial number of transmissions is unclear, even in settings with a quick and vigorous public health response to new outbreaks. Gomes et al. (*4*) performed simulations of Ebola outbreaks in each of 220 countries by first estimating the risk of Ebola being exported from Guinea, Liberia, or Sierra Leone by international travelers and then simulating a stochastic Ebola transmission model conditioned on an importation. The model incorporated Ebola transmission from infected persons in the community and hospital settings and from recently deceased Ebola patients. Assumptions used in the model were that only community transmissions are relevant outside of Africa and that transmissions occur at rates corresponding to containment measures already in place. Gomes et al. provided no explicitly numerical probabilities of large outbreaks per importation, but their simulations apparently produced <100 cases in each country. 

In another study, Rainisch et al. (*18*) calculated the estimated number of beds required to treat Ebola patients in the United States by using estimates of importation frequency and subsequent transmission. These researchers reported a high estimate of 7 beds (95% CI 2–13) required at any 1 time; they also provided no numerical probabilities for their estimates.

In our study, we use a branching process model to estimate the probability distribution of outbreak sizes resulting from the introduction of an Ebola case to a new country where the reproductive number *R* (i.e., expected number of transmissions per case) would likely be quickly, if not immediately, reduced to <1. In this scenario, theory from subcritical branching processes (*19*), also known as mortal branching processes (*20*), guarantees that an outbreak will eventually die out, although perhaps not before a substantial number of transmissions occur. In the modeling literature, outbreaks that die out on their own have been called minor outbreaks (*21*) or stuttering chains (*22*). Such branching process models have been used to estimate transmission parameters in the context of emerging (*22*,*23*) or reemerging (*19*–*21*,*24*) infectious diseases. However, unlike other studies, we used the outbreak final size distribution equations derived from branching process theory to calculate the risk for a large Ebola outbreak under the assumptions of immediate and delayed transmission control after an importation.

## Materials and Methods

We first gathered transmission data for all Ebola patients who were documented in the ongoing West Africa outbreak and who spent all or part of their infectious periods in a country other than Guinea, Sierra Leone, or Liberia. We next fit the negative binomial distribution to the transmission data and to various data subsets according to patients’ circumstances. We also applied the theory of branching processes with a negative binomial offspring distribution to estimate the probability that new introductions would lead to outbreaks exceeding various sizes. We then organized these estimates under 2 scenarios of transmission control: immediate and delayed. For each scenario, we tested the effects of different levels of variability in transmission.

In the data-gathering step, we compiled information for 56 documented Ebola patients (Table 1) who spent all or part of their infectious period in 1 of 12 countries other than Guinea, Sierra Leone, or Liberia. We broke these data into 3 subgroups: patients who traveled to 1 of the 12 countries during their incubation or symptomatic period, patients deliberately evacuated from West Africa for treatment, and patients who acquired infection in the new country after an introduction of the virus.

**Table 1:** Characteristics of Ebola case-patients reported outside Guinea, Sierra Leone, and Liberia*

Case-patients	Year and month	Country	Circumstance of infection	No. transmissions
1	2014 Jul	Nigeria	Imported by traveler	13
2–4	2014 Jul–Aug	Nigeria	Locally acquired	1
5–19	2014 Jul–Aug	Nigeria	Locally acquired	0
20	2014/Aug	Nigeria	Locally acquired	3
21–23	2014/Aug	Spain, United Kingdom, Germany	Evacuated	0
24	2014/Aug	Senegal	Imported by traveler	0
25–27	2014/Aug–Sep	United States	Evacuated	0
28	2014/Sep	France	Evacuated	0
29	2014/Sep	United States	Imported by traveler	2
30	2014/Sep	Spain	Evacuated	1
31–32	2014/Oct	United States	Locally acquired	0
33	2014/Oct	Spain	Locally acquired	0
34–35	2014/Oct	Germany	Evacuated	0
36	2014/Oct	Norway	Evacuated	0
37	2014/Oct	United States	Imported by traveler	0
38	2014/Oct	Mali	Imported by traveler	0
39–40	2014/Oct–Nov	United States	Evacuated	0
41	2014/Nov	France	Evacuated	0
42	2014/Nov	Mali	Imported by traveler	5
43–44	2014/Nov	Mali	Locally acquired	1
45–49	2014/Nov	Mali	Locally acquired	0
50–52	2014/Nov	United States, Switzerland, Italy	Evacuated	0
53	2014/Dec	The Netherlands	Evacuated	0
54	2014/Dec	United Kingdom	Imported by traveler	0
55–56	2015/Mar	United Kingdom, United States	Evacuated	0

*These data are published and publicly available as of April 24, 2015. Month/year is when patients were transferred or diagnosed. References by country: Nigeria (*5**,**6*); Spain (*15**,**16*); United Kingdom (*14**,**15*); Senegal (*12*);United States (*7**,**8**,**13**,**15*); Mali (*9**–**11*); other countries *(**15*).

We fit the transmission data from patients within subgroups to the negative binomial distribution with mean *R* and dispersion parameter *k*, which characterizes individual variation in transmission, including the likelihood of superspreading events (i.e., when infected persons disproportionately transmit the virus to others) (*25*). High values of *k* produce low variability and a low probability of superspreading; *k* approaching infinity leads to a Poisson distribution, which arises when all infected persons have an equal expected number of contacts and an equal probability of transmission per contact. The value *k* = 1 corresponds to a geometric distribution, which arises when the duration of the infectious period varies among infected persons according to an exponential distribution, as is generally assumed by differential equation models; otherwise, the infected persons are homogeneous. Values of *k* <1 produce more highly dispersed models, which occur when infected persons vary substantially in numbers of susceptible contacts or in probabilities of transmission per contact (*25*). Occurrence of high variability leads to a higher probability of superspreading.

We estimated the parameters *R* and *k* for each data subset by using the method of moments, which calculates the parameter values that produce the exact mean and variance exhibited by the data (i.e., a single estimate for *R* and *k*). We calculated associated confidence intervals by using a bias-corrected percentile method (*26*) on random samples of the data. We also used a Kolmogorov–Smirnov test (*27*) to assess goodness of fit (Technical Appendix).

We then applied theory from branching process models, which use a discrete probability distribution, or offspring distribution, to specify the number of transmissions resulting from each infected person in a chain of transmissions. We used the negative binomial distributions fitted to the Ebola transmission data for the offspring distribution. Because our *R* estimates differed depending on the data subgroups representing each infected person’s circumstances, we examined models representing an immediate control scenario and a delayed control scenario, each with 2 levels of control, for a total of 4 combinations of parameters.

In the immediate control scenario, the initial infected person or persons and any subsequent infected persons transmit infection according to the same distribution with *R*<1. In the delayed control scenario, the initially introduced infected person or persons have a higher expected number of transmissions (*R*_0_, the initial or basic reproductive number) than the number expected to be transmitted from subsequent cases (*R*_c_, the postcontrol reproductive number). The delayed control scenario can occur when an infected traveler arrives in a new country during the incubation or symptomatic period and has contact with others before the person has been identified as infected or when the person is treated in a facility that is not fully prepared or experienced in handling an Ebola patient, but any subsequent cases are identified quickly and handled more effectively by a well prepared facility. For controlled patients in either scenario, we examined 2 different levels of control; these levels are represented by 2 values of the postcontrol reproductive number: 1 derived from the average number of transmissions from evacuated infected persons and 1 from the average number of transmissions from persons who acquired infection locally. Because the *k* values calculated from the fitting of transmission data ranged widely, we applied a series of 3 test values (*k* = 0.1, 1, and 10) to each scenario to determine the effect of variability on the outcomes.

For each scenario and each set of parameter assumptions, the probability distribution of final outbreak sizes according to branching process theory can be calculated (online Technical Appendix) as examples of Lagrangian distributions (*28*,*29*). For each parameter combination, we used these equations to calculate the probability of exceeding given outbreak sizes, up to the size expected to be exceeded with a probability of ≈0.01%. To compare different scenarios, we calculated the probability of exceeding 10 and 100 total transmissions and worst-case outbreak sizes (i.e., the number of transmissions expected to be exceeded after 1% and 0.01% of introductions of Ebola). Although we show only results calculated with the assumption of 1 initial patient, the equations we provide (online Technical Appendix) generalize to any number of initial patients and can be used in situations for which multiple introductions might be of interest.

## Results

Certain subgroups of patients within the dataset produced substantially different *R* estimates (Table 2). For patients who traveled to 1 of the 12 countries during their incubation or symptomatic period, we calculated *R* = 2.9 transmissions per patient; for patients deliberately evacuated from West Africa for treatment, *R* = 0.05; for patients who acquired infection in the new country after an introduction, *R* = 0.3. Estimates of *k* produced wide CIs within subgroups, from values <1, which are consistent with a highly dispersed distribution, to large values, which are consistent with a Poisson distribution.

**Table 2:** Summary of Ebola data and parameter estimates*

Patient group	No.	Transmissions	*R* estimate (90% CI)	*k* estimate (90% CI)
All	56	29	0.5 (0.2–1.0)	0.09 (0.03–0.2)
Traveler	7	19	2.9 (0.6–6.1)	0.4 (0.2–1.3)
Evacuated patient	20	1	0.05 (0–0.1)	∞
Patient with locally acquired Ebola	29	9	0.3 (0.1–0.5)	0.5 (0.2–∞)

*Cases were included if the patient spent any of the infectious period in a country other than Guinea, Liberia, or Sierra Leone. The 56 patients are split into 3 mutually exclusive subgroups, depending on the patients’ circumstances. Parameters *R* and *k* of the negative binomial distribution are the reproductive number and dispersion parameter, respectively. Goodness of fit was not rejected by a Kolmogorov–Smirnov test (p>0.6 in all cases).

**Figure 1:**
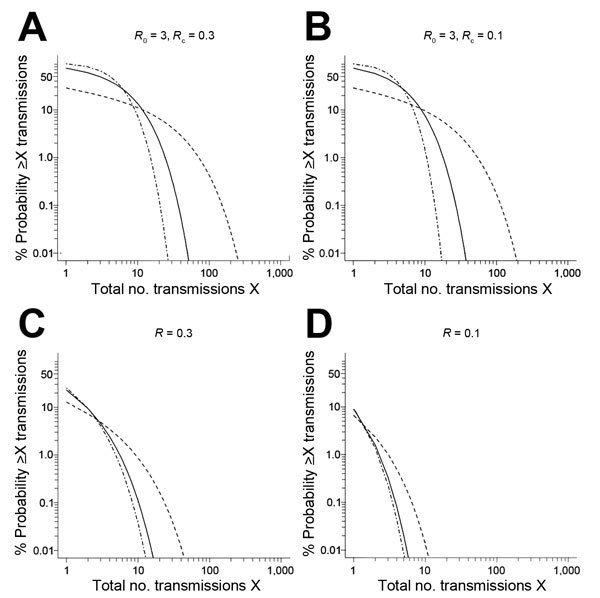
Exceedance risk curves for total number of transmissions in an Ebola outbreak following a single-case introduction. Solid lines, *k* = 1; dashed lines, *k* = 0.1; dash-dot lines, *k* = 10. A) *R*_0_ = 3 for initial case, assumed to be traveler during incubation or symptomatic period; and *R*_c_ = 0.3 for subsequent cases, assumed to be locally acquired cases in countries other than Guinea, Sierra Leone, or Liberia. B) *R*_0_ = 3 for initial case, assumed to be patients evacuated for treatment; and *R*_c_ = 0.1 for subsequent cases. C) *R* = 0.3 for all cases. D) *R* = 0.1 for all cases.

Assuming *R*_0_ = 3, estimated on the basis of traveler-imported cases, and *R*_c_ = 0.3, estimated on the basis of locally acquired cases, the chance of an outbreak with >10 transmissions after a single introduction ranges from ≈7%–13% across the 3 assumed values for *k*: 0.1, 1, and 10 (Figure 1, panel A). The chance of an outbreak with >100 transmissions is negligible (<0.01%) for *k* = 10 and *k* = 1 but rises to ≈0.4% under a high variability assumption of *k* = 0.1 because of increased likelihood of superspreading. We considered the effect of 2 ways to reduce the risk from those results: decreasing *R*_c_ to approach the level of control achieved among evacuated patients (*30*) and eliminating the initial high-average transmission step to reflect preidentification of the initial infected traveler.

When *R*_0_ = 3 but *R*_c_ is decreased to 0.1 (Figure 1, panel B), the chance of >10 transmissions is 1%–10%. The chance of >100 transmissions is <0.01% for *k* = 10 and *k* = 1 and 0.2% with high variability. Assuming that the initial patient is identified and that transmission is controlled (*R* = 0.3) (Figure 1, panel C) causes a much more substantial decrease in outbreak risk, to a range of 0.04%–1% for >10 transmissions and <0.01% for >100 transmissions, even with high variability. Assuming *R* = 0.1 for all patients causes >10 transmissions to be very unlikely, with a 0.01% chance even with high variability (Figure 1, panel D).

**Figure 2:**
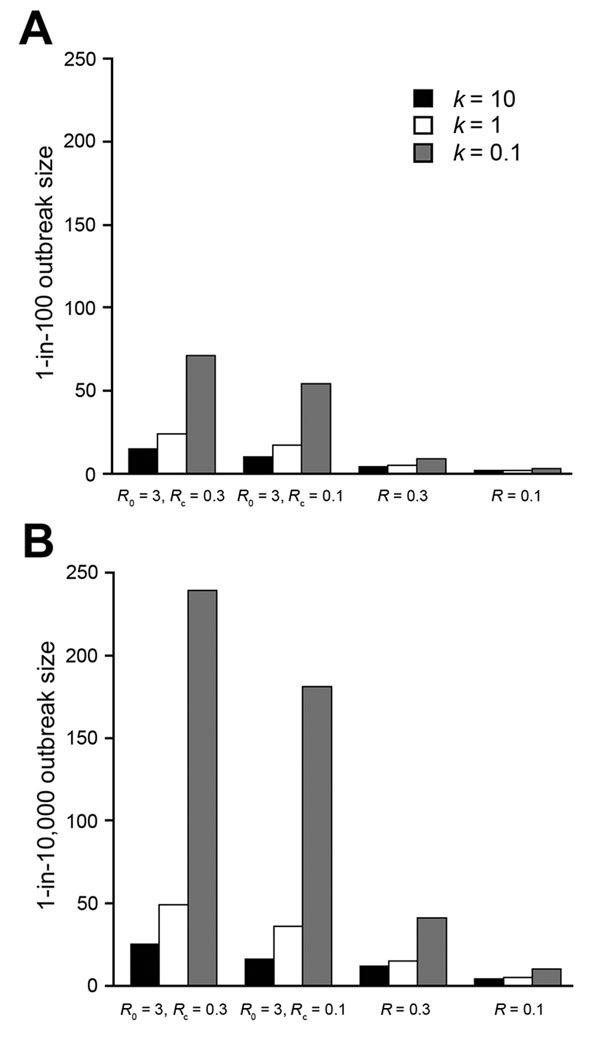
Comparison of worst-case Ebola outbreak sizes after a single-case introduction under different scenarios. Comparisons of the outbreak size expected to be exceeded after A) 1% of introductions and B) 0.01% of introduction of a single initial case, under different assumptions for the reproductive number *R* and dispersion parameter *k*. In all cases, higher transmission variability (lower *k*) leads to higher worst-case estimates. From the *R*_0_ = 3, *R*_c_ = 0.3 case, reducing *R*_c_ to 0.1 for cases after the initial case has less effect than reducing the initial case *R*_0_ to 0.3. Reducing both the initial and subsequent cases’ *R* to 0.1 has a synergistic effect.

In addition, we compared the 4 scenarios and considered worst-case outbreaks at 2 probability levels: the outbreak level estimated by the model to be exceeded in 1% of introductions (Figure 2, panel A) and the outbreak level estimated by the model to be exceeded in 1 in 10,000 introductions (Figure 2, panel B). The effect of identifying the initial patient is stronger than the effect of reducing *R*_c_, but the combination produces a synergistic effect. For example, at moderate variability (*k* = 1), the 0.01% worst-case outbreak size when *R*_0_ = 3 and *R*_c_ = 0.3 (49 total transmissions) is reduced to 73% of that value (36 total transmissions) when *R*_c_ is reduced to 0.1. The worst-case outbreak size is reduced to 31% (15 total transmissions) when the initial patient is identified. Reducing *R*_c_ (i.e., postcontrol average number of transmissions per patient) and identifying the initial patient together decrease transmission size to 10% of the worst-case value (5 total transmissions), which is greater than the expected reduction (to 22%) if each intervention was conducted independently. The worst-case risk reduction is greatest under the assumption of high transmission variability (*k* = 0.1); the 0.01% worst-case outbreak size is reduced from 239 total transmissions to 4% or 10 total transmissions when both intervention assumptions are applied.

We also explored the sensitivity of exceedance probabilities to additional values of *R* and *k* (Technical Appendix Figure) and showed nuances of how higher variability can simultaneously increase the probability of the best-case scenario (no transmissions) and of worst-case scenarios (e.g., superspreading, which can lead to large outbreaks).

## Discussion

The outbreak size distributions produced by our models are comparable to those of Gomes et al. (*4*), although their results also encompassed frequencies of case exportations from Guinea, Sierra Leone, or Liberia into each particular country, which our analysis did not include. None of their simulations appears to have produced >100 cases in any particular country, indicating that our scenarios result in more pessimistic outcomes. For example, our delayed-control, high-variability (*k* = 0.1) scenario produced a 0.4% probability of >100 transmissions after a single introduction. The assumed transmission probabilities of Gomes et al. appear to be more comparable to our immediate-control and lower-variability scenarios.

Our estimates also assign greater potential probabilities of large outbreaks than those provided by Rainisch et al. (*18*), whose highest estimate of the number of beds required at a given time to treat Ebola patients in the United States was 7 (95% CI 2–13). The difference between this result and ours is farther widened because their result includes the possibility of multiple simultaneous introductions caused by a cluster of infected travelers, whereas our results were based on a single introduction. Their worst-case estimate is lower than ours because Rainisch et al. assumed a maximum of 2 additional cases caused by secondary transmission per imported case, whereas our fitted distributions of possible transmissions reach >2, sometimes with substantial probability. For example, in our scenario of a single unidentified traveler’s Ebola introduction with *R*_0_ = 3 and subsequent cases transmitting with *R*_c_ = 0.3 and with moderate transmission variability (*k* = 1), our model estimates a 50% chance of >2 transmissions occurring after a single introduction. However, under the assumption of immediate transmission control (*R*_c_ = 0.1), our model estimates <1% chance of >2 transmissions.

The contrasting relationships between the parameter *k* and different measures of outbreak risk reflect the unpredictable outcome of high variability in transmission: a low frequency of outbreaks after a new introduction but a relatively high probability of an explosive outbreak when an outbreak occurs. This situation was seen in countries experiencing introduced cases of severe acute respiratory syndrome, for which estimated values of *k* were ≈0.1 (*25*). A recent study (*31*) produced a similar estimate (*k* = 0.18; 95% CI 0.10–0.26) when the negative binomial distribution was fitted to data from large Ebola transmission chains in Guinea (*32*); this result suggests that the high variability assumption may be appropriate, but whether or not the assumption of high variability is an appropriate characterization for potential Ebola outbreaks in new countries is unclear. Attempting to estimate *k* by using transmission data (Table 1) produced wide ranges of uncertainty. However, the Ebola case that resulted in 13 transmissions in Nigeria suggests that assuming a low value of *k*, at least for the delayed control scenario, is justified. In Nigeria, the average number of transmissions from those 13 and all subsequent cases was <0.4. This low average number of transmissions was assisted by health authorities’ rapid implementation of control measures (*5*). Little evidence exists for high transmission variability from these or other locally acquired or medically evacuated Ebola cases. 

Our framework quantitatively characterizes worst-case Ebola outbreaks resulting from an introduced Ebola case to a region with relatively effective control measures. Our results can be used by public health officials engaging in risk-benefit analyses of potential decisions affecting Ebola case introductions, such as decisions to evacuate infected or potentially infected persons from West Africa, policies on travel surveillance measures, and strategies for handling identified importations. Initial public health assessments at the local level include the risk of importing a case to that geographic area (*4*,*18*,*33*,*34*), the cost versus benefit of identifying potential cases through traveler screening (i.e., in major international ports of arrival), or surveillance in health care facilities (that serve populations at risk from travel or exposure). Either way, the tried and tested methods of early detection and isolation appear to be of primary importance in controlling ongoing Ebola outbreaks in West Africa (*35*) or potential new outbreaks caused by imported cases elsewhere.

Our framework also provides a simple method to quantify the individual and synergistic effects of different control strategies. Our results stress the paramount importance of surveillance measures to identify international travelers who may have been recently exposed to Ebola virus because a higher reproductive number from initially introduced cases can drastically increase the risk of a large outbreak, even if effective control measures are immediately put in place to reduce transmission from subsequent cases. Surveillance, combined with measures to reduce transmission from local cases to the low average achieved among evacuated cases (*30*), can reduce the probability of all but a handful of transmissions to negligible levels.

**Technical Appendix.** Detailed background and methods for quantitative analyses, equations used to generate model results, and results from additional sensitivity analyses.
