# Supplementary material for: Estimates of Outbreak Risk from New Introductions of Ebola with Immediate and Delayed Transmission Control
Source: Emerg Infect Dis. 2015 Aug;21(8):1402–8. doi: 10.3201/eid2108.150170 (PMC4517734; doi:10.3201/eid2108.150170)
Supplement: Supplementary file 1 — Technical Appendix. Detailed background and methods for quantitative analyses, equations used to generate model results, and results from additional sensitivity analyses. [file 15-0170-Techapp-s1.pdf]

Article DOI: http://dx.doi.org/10.3201/eid2108.150170

Estimates of Outbreak Risk from New Introductions of Ebola with Immediate and Delayed Transmission Control

Technical Appendix

Additional Methods, Equations, and Results

To fit the negative binomial model to each dataset, we used a method-of-moments estimator, which calculates R and k values that produce the exact mean and variance exhibited by the data. To estimate 90% confidence intervals, we ran 1 million nonparametric bootstrap resamples of each dataset, with replacement, and recalculated the R and k estimates for each resample. Then we used a bias-corrected percentile method (*1*) to construct the confidence intervals. We used the 1-sample Kolmogorov–Smirnov test, adapted for discrete variables (*2*), to assess goodness of fit, and the null hypothesis that each dataset was generated from the given negative binomial distribution was not rejected (P >0.6 in all cases).

To model outbreaks stemming from case introductions, we first assumed a branching process in which the number of transmissions from each infected person is independent and identically distributed according to a discrete probability distribution governed by a probability-generating function (pgf) $f(s)$. The probability, p_{nz}, that n independent infected persons produce a total of z transmissions, is the zth coefficient of the power series representation of $[f(s)]^n$, which can be extracted by calculating

$$p_{nz} = \frac{1}{z!}\left[\frac{d^z}{ds^z}[f(s)]^n\right]_{s=0}.$$

We are interested in the probability that a branching process that goes extinct (a minor outbreak or stuttering chain) includes a given *total* number of cases, X, over all generations, including the initial case(s) in the total. When there is a single initial case, this value is governed by a pgf, $g(s)$, satisfying the following equation:

$$g(s) = sf\big(g(s)\big).$$

Solving for the coefficients of the power series representation of $g(s)$ can be achieved by using a Lagrange expansion, which results in the following (*3*):

$$P(X = x) = \frac{1}{x!}\left[\frac{d^x}{ds^x} g(s)\right]_{s=0} = \frac{1}{x!}\left[\frac{d^{x-1}}{ds^{x-1}} \big(f(s)\big)^x\right]_{s=0}.$$

Probability distributions of this form have been named *basic Lagrangian distributions* (*4*).

When the number of initial cases is a random variable with pgf $f_0(s)$, then further Lagrange expansion results can be used to obtain the result,

$$P(X = x) = \frac{1}{x!}\left[\frac{d^{x-1}}{ds^{x-1}} \left\{\big(f(s)\big)^x f_0{}'(s)\right\}\right]_{s=0}.$$

In the case that the number of initial cases is fixed at n, we have $f_0(s) = s^n$, leading to a *delta Lagrangian distribution* (reverting to basic when $n = 1$); otherwise, we have a *general Lagrangian distribution* (*4*).

Example distributions have been generated by substituting pgf's $f(s)$ and $f_0(s)$ of several different discrete probability distributions into the above equations (*3*). When $f(s)$ is the pgf of the negative binomial distribution with mean R and dispersion parameter k:

$$f(s) = \left(1 + \frac{R}{k}(1 - s)\right)^{-k},$$

we have

$$p_{nz}(R, k) = \frac{\Gamma(kn + z)}{z!\,\Gamma(kn)}\left(\frac{R}{R + k}\right)^{z}\left(\frac{k}{R + k}\right)^{kn},$$

where Γ represents the gamma function. Here, p_{nz} is the probability distribution for the number of transmissions z from the initial patient(s) only. The distribution for the total number of patients x over an entire stuttering chain starting with n initial patients is

$$q_{nx}(R, k) = \frac{n}{x}\frac{\Gamma(kx + x - n)}{(x - n)!\,\Gamma(kx)}\left(\frac{R}{R + k}\right)^{x-n}\left(\frac{k}{R + k}\right)^{kx},$$

where we have taken the "neg. binomial-delta" formula in Consul and Shenton (*3*) and replaced their parameterization of the negative binomial distribution with the one above.

Blumberg and Lloyd-Smith (*5*) derived an equivalent result, although only for the scenario $n = 1$, without using the Lagrange expansion technique. The result above shows that a generalization of their approach would yield the following relationship:

$$q_{nx}(R,k) = \frac{n}{x} p_{x,x-n}(R,k).$$

This equation gives the intuition that an outbreak with x total patients means that those x patients produced a total of exactly $x - n$ transmissions, and the probability of that occurring from x independent patients must be adjusted by the fraction (n / x) to account for the fact that the transmissions must occur in an order that produces a valid transmission chain (*5*). This result was also described by Becker (*6*), who derived the final size distribution when the offspring distribution is expressed as a generalized power series distribution, of which the negative binomial distribution is a special case.

Next, we consider a scenario in which n initially infected persons transmit according to a negative binomial distribution with parameters (R_0, k_0) and any and all subsequent persons transmit according to a different negative binomial distribution with parameters (R_c, k_c). The probability r_{nx} of an outbreak of total size x (including the n initial patients) is

$$r_{nx}(R_0,k_0,R_c,k_c) = \begin{cases} p_{n0}(R_0,k_0), & x = n \\ \sum_{z=1}^{x-n} p_{nz}(R_0,k_0)\, q_{z,x-n}(R_c,k_c), & x > n. \end{cases}$$

The sum in this equation can be expressed by using a hypergeometric function, as in Consul and Shelton (*3*) (general Lagrangian distribution for the double-negative binomial case) for the case $n = 1$, but we found the above expression to be more convenient for calculations.

To calculate the probability of x or more transmissions, we evaluated

$$1 - \sum_{m=n}^{n+x-1} q_{nm}(R,k),$$

or

$$1 - \sum_{m=n}^{n+x-1} r_{nm}(R_0, k_0, R_c, k_c).$$

Although we have limited our study to scenarios in which R<1 or R_c<1, these exceedance probability equations are valid for all positive values of R or R_c.

We further explored the effects of assuming different values of R and k in the initially controlled scenario by varying R from 0 to 1 and k from 0.01 to 100 and plotting the resulting probabilities of exceeding 1, 5, 10, and 100 total cases in an outbreak seeded by 1 person (Technical Appendix Figure, panels A, B, C, D). These results show contrasting interpretations of the parameter k. Although increasing R always increases exceedance probabilities, the effect of increasing k is not always the same, as higher variability (lower k) increases the probability of both below-average and above-average transmissions. For example, with R fixed at a specific value, a lower value of k decreases the probability of $\geq$1 transmissions (Technical Appendix Figure, panel A); that is, lower k increases the probability that the initial case will not transmit, which is the best-case scenario for a country experiencing an introduction. However, a lower value of k also increases the probability of worst-case scenarios for certain values of R, for example, the probability of $\geq$10 transmissions for R = 0.1 (Technical Appendix Figure, panel C) or the probability of $\geq$100 transmissions for R = 0.6 (Technical Appendix Figure, panel D).

References

1. DiCiccio T, Efron B. Bootstrap confidence intervals. Stat Sci. 1996;11:189–228. http://dx.doi.org/10.1214/ss/1032280214

2. Arnold TB, Emerson JW. Nonparametric goodness-of-fit tests for discrete null distributions. R J. 2011;3/2:34–9.

3. Consul PC, Shenton LR. Use of Lagrange expansion for generating discrete generalized probability distributions. SIAM J Appl Math. 1972;23:239–48. http://dx.doi.org/10.1137/0123026

4. Consul PC, Famoye F. Lagrangian probability distributions. Boston: Birkhauser; 2006.

5. Blumberg S, Lloyd-Smith JO. Inference of R0 and transmission heterogeneity from the size distribution of stuttering chains. PLOS Comput Biol. 2013;9:e1002993. PubMed http://dx.doi.org/10.1371/journal.pcbi.1002993

6. Becker N. On parametric estimation for mortal branching processes. Biometrika. 1974;61:393–9. http://dx.doi.org/10.1093/biomet/61.2.393

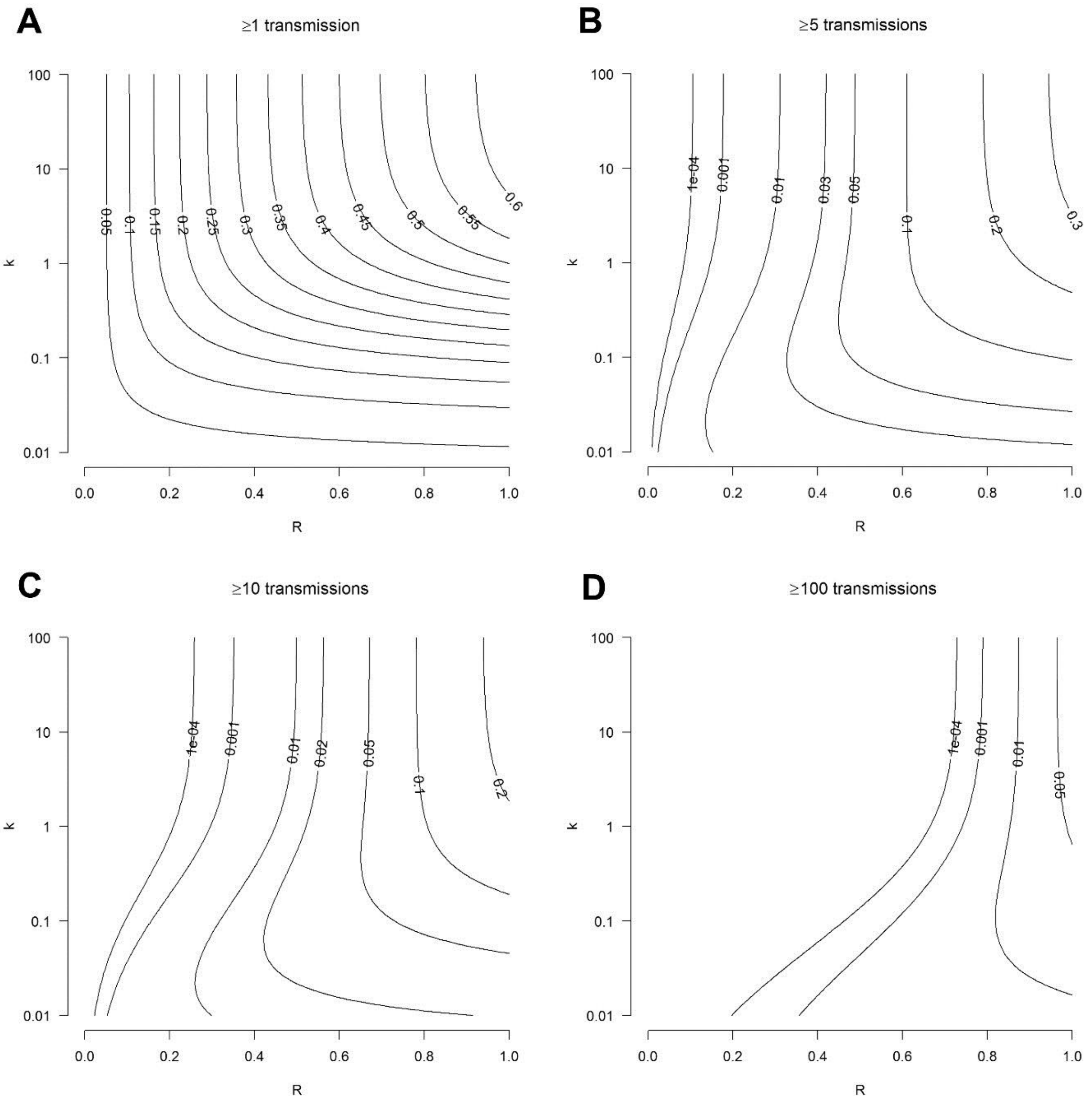

Technical Appendix Figure. Exceedance probability contours showing *R* and *k* sensitivity. Probability contours in (*R*, *k*) parameter space with the assumption of a single initial patient. *R* is the reproductive number (i.e., average transmissions from each patient, including the initial patient), and *k* is the negative binomial dispersion parameter; a lower *k* corresponds to higher individual variability in transmission. A) Probability of ≥1 transmissions from the initial case; B) probability of ≥5 transmissions over the entire outbreak; C) probability of ≥10 transmissions over the entire outbreak; D) Probability of ≥100 transmissions over the entire outbreak.
